# Network pharmacological analysis of active components of Xiaoliu decoction in the treatment of glioblastoma multiforme

**DOI:** 10.3389/fgene.2022.940462

**Published:** 2022-08-15

**Authors:** Ji Wu, Xue-Yu Li, Jing Liang, Da-Lang Fang, Zhao-Jian Yang, Jie Wei, Zhi-Jun Chen

**Affiliations:** ^1^ Department of Neurosurgery, Affiliated Hospital of Youjiang Medical University for Nationalities, Baise, China; ^2^ Department of Pediatrics, The Second Affiliated Hospital of Xinjiang Medical University, Urumchi, China; ^3^ Department of Breast and Thyroid Surgery, Affiliated Hospital of Youjiang Medical University for Nationalities, Baise, China; ^4^ Department of Neurosurgery, Red Cross Hospital of Yulin City, Yulin, China; ^5^ Department of Hematology, People’s Hospital of Baise, Baise, China

**Keywords:** network pharmacology, Xiaoliu decoction, glioblastoma multiforme, traditional Chinese medicine, system analysis, treatment

## Abstract

**Background:** Glioblastoma multiforme (GBM) is the most aggressive primary nervous system brain tumor. There is still a lack of effective methods to control its progression and recurrence in clinical treatment. It is clinically found that Xiaoliu Decoction (XLD) has the effect of treating brain tumors and preventing tumor recurrence. However, its mechanism is still unclear.

**Methods:** Search the Traditional Chinese Medicine System Pharmacology Database (TCSMP) for efficient substances for the treatment of XLD in the treatment of GBM, and target the targeted genes of the effective ingredients to construct a network. At the same time, download GBM-related gene expression data from the TCGA and GTEX databases, screen differential expression bases, and establish a drug target disease network. Through bioinformatics analysis, the target genes and shared genes of the selected Chinese medicines are analyzed. Finally, molecular docking was performed to further clarify the possibility of XLD in multiple GBMs.

**Results:** We screened 894 differentially expressed genes in GBM, 230 XLD active ingredients and 169 predicted targets of its active compounds, of which 19 target genes are related to the differential expression of GBM. Bioinformatics analysis shows that these targets are closely related to cell proliferation, cell cycle regulation, and DNA synthesis. Finally, through molecular docking, it was further confirmed that Tanshinone IIA, the active ingredient of XLD, was tightly bound to key proteins.

**Conclusion:** To sum up, the results of this study suggest that the mechanism of XLD in the treatment of GBM involves multiple targets and signal pathways related to tumorigenesis and development. This study not only provides a new theoretical basis for the treatment of glioblastoma multiforme with traditional Chinese medicine, but also provides a new idea for the research and development of targeted drugs for the treatment of glioblastoma multiforme.

## Introduction

Human cancer has become a main cause of death, and glioma, which can be divided into low-grade glioma and high-grade glioma, is the most frequent main central nervous system malignant tumor. Glioblastoma multiforme (GBM) is the most common primary malignant brain tumor, accounting for 14.5% of all tumors and 48.6% of malignant tumors. At present, there is no cure for GBM, and GBM patients have a median survival duration of fewer than 8 months. (Diwanji et al., 2017; Ostrom et al., 2020). Despite the development of novel GBM therapies, surgery, radiation, and chemotherapy remain the most effective treatments (Rusthoven et al., 2017; Tamimi and Juweid, 2017). Surgical therapy should be first explored in patients with primary brain tumors without distant metastases. However, it is often impossible to completely remove the tumor without damaging the nervous system of patients. As a result, whole-brain radiotherapy or stereotactic radiotherapy is needed after the operation (Chaichana et al., 2014; Mengue et al., 2020). For some GBM with distant metastasis, chemotherapy is an alternative treatment. Additionally, molecular targeted therapy is expected to become a breakthrough in cancer treatment. However, due to the presence of the blood–brain barrier, the roles of chemotherapy and molecular targeted therapy in the treatment of GBM have not been further confirmed (Bastien et al., 2015; van Tellingen et al., 2015; Rusthoven et al., 2017). Therefore, it is necessary to explore natural traditional Chinese medicine (TCM) monomers, which can effectively target GBM by penetrating the blood–brain barrier.

TCM has unique tumor prevention and treatment effects, and it is an important part of comprehensive tumor treatment. A large number of clinical studies have demonstrated that TCM can be combined with surgery, radiotherapy and chemotherapy to achieve better results, prevent tumor progression, alleviate clinical symptoms and reduce adverse reactions of radiotherapy and chemotherapy, thereby improving the quality of life and prognosis of patients (The Lancet Oncology, 2015). For example, Xiaoliu decoction (XLD) has been widely used in China and achieved definite curative effects in colorectal cancer, liver cancer, pancreatic cancer, non-small cell lung cancer and glioma (Fu and Xia, 2004; Wang et al., 2004; Hou et al., 2018). The recommended formula mainly includes Baihuasheshecao (15 g), baizhi (10 g), Baizhu (15 g), banxia (10 g), Banzhilian (15 g), chenpi (10 g), Chuanxiong (10 g), Danshen (15 g), Fuling (15 g), Gancao (6 g), Gouteng (10 g) and Yanhusuo (10 g). Table 1 shows the complete scientific species names (Latin binomial nomenclature) of all components of XLD obtained from TCMID, a comprehensive database of TCM.

**TABLE 1:** The Chinese names of the components of each herbal medicine of XLD and their corresponding Latin names.

Chinese pinyin name	Latin name
baizhi	A. Dahurica (Fisch.) Benth. Et Hook
Baizhu	Atractylodes Macrocephala Koidz
banxia	Arum Ternatum Thunb
Banzhilian	Scutellariae Barbatae Herba
chenpi	Citrus Reticulata
Chuanxiong	Chuanxiong Rhizoma
Danshen	Radix Salviae
Fuling	Poria Cocos (Schw.) Wolf
Gancao	licorice
Gouteng	Uncariae Ramulus Cumuncis
Yanhusuo	Corydalis Rhizoma
Baihuasheshecao	Hedyotis Diffusae Herba

Among TCMs, licorice is the core ingredient of XLD. LICRICELICRICE (*Glycyrrhiza uralensis*) was first found in Shennong Materia Medica, the oldest pharmacopeia in China. Licorice is widely used in clinical prescriptions of TCM. Modern pharmacological studies have shown that *Glycyrrhiza uralensis* has a variety of biological activities, such as anti-tumor, anti-virus, anti-inflammatory, antioxidant, immune regulation, liver protection and nerve protection. The effects of several components in *Glycyrrhiza uralensis* have also been studied in glioblastoma multiforme. Glycyrrhizin (ALA) is a natural chalcone extracted from *Glycyrrhiza uralensis*. It induces mitochondrial dysfunction in glioblastoma multiforme stem cells (GSCs) and further activates the mitochondrial apoptosis signaling pathway, resulting in cell death *in vitro*. Tanshinone IIA is the main active component of *Salvia miltiorrhiza*. Recent studies have shown that it inhibits the proliferation, migration and invasion of GBM through miR-16-5p/Talin-1 (Ryu et al., 2012; Yang et al., 2014; You et al., 2020). Although some of the ingredients in XLD have been reported, there is limited knowledge regarding its composition and therapeutic effects. The purpose of this paper was to analyze the active components, potential key targets and biological pathways of XLD. The current study of traditional Chinese medicine for the treatment of glioblastoma multiforme mainly focuses on the study of the simple mechanism of one medicine on the development of tumour, while XLD is currently used in clinical practice, but its specific mechanism of action and core active ingredients for the treatment of glioblastoma multiforme are unknown to us, therefore, it is necessary to explore it, we used a large number of tissues from TCGA and GTEX database The study was conducted to obtain differentially expressed genes for glioblastoma multiforme and to systematically analyse the mechanism of XLD formulae for the treatment of glioma through a network pharmacology approach. In addition to this, the active ingredients in XLD were restricted to cross the blood-brain barrier, and ultimately, molecular docking was used to verify that these active ingredients could bind to the target genes. This will provide a theoretical basis for the subsequent development of small molecule drugs for the targeted treatment of glioblastoma multiforme. This study provides useful information to understand the biological process of XLD in GBM and offers new therapeutic options for the treatment of GBM.

## Materials and methods

### Data preparation

#### Searching for active ingredients of XLD

The active ingredients of XLD are downloaded (https://tcmsp-e.com/) from the traditional Chinese Medicine system Pharmacology (TCMSP) Database (Ru et al., 2014), which contains many traditional Chinese medicine entries, drug disease networks and drug target networks. A large number of herbal information is available from the TCMSP database, including composition, molecular name, molecular weight (MW), drug similarity (DL), human oral bioavailability (OB), half-life (HL), water partition coefficient (AlogP), number of hydrogen-bonded donors and receptors (Hdon/Hacc), Caco-2 permeability (Caco-2) and blood-brain barrier (BBB). Oral bioavailability (OB) is one of the most important pharmacokinetic characteristics of oral drugs to evaluate the drug delivery efficiency to systemic circulation. Its value is calculated from the OBioavail 1.1 model developed by the research group in the previous stage, and the (Ru et al. is calculated. 2014). Oral bioavailability indicates the percentage of efficacy that can be produced by a unit of oral dose, which is a key index to determine the drug properties of active molecules. Drug-like drugs are used to evaluate the possibility of compounds becoming drugs, and only the molecules with higher OB and DL may have good pharmacological activity. The average DL of drugs in the reference Drug Bank database is 0.18. Studies have shown that compounds with BBB < -0.3 are considered to be non-penetrating (BBB-), moderate penetration (BBB ±) from-0.3 to +0.3, and strong penetration (BBB+) from 0.3 to +0.3. Therefore, the ingredient with oral bioavailability standard ≥30% and DL ≥ 0.18, BBB > -0.3 was regarded as active ingredient (Wang et al., 2015).

#### (PPI) analysis of protein-protein interaction

After screening the active components of XLD through the TCMSP database, the active components are uploaded to PubChem (https://www.ncbi.nlm.nih.gov/), a multi-functional Web server for exploring the relationship between pharmacological and chemical structures based on molecular 3D similarity methods (Wang et al., 2017b). We searched the ChemMapper database for the prediction targets of each active component in XLD, and screened them according to the criteria that the 3D structural similarity was higher than 1.0 and the prediction score was higher than 0, and found the two-dimensional structure of the active component (Liu et al., 2010). In addition, Uniprot (https://www.uniprot.Org) are used to standardize the prediction targets (The UniProt Consortium, 2018), and express them in the form of gene ID. In order to improve the credibility of the target, the STITCH database (https://cn.string-db.org/), which is commonly used to search and predict the interaction between the compound and the target protein, is also used to predict the target. Finally, the Venn diagram is made, and the intersection of the two databases is regarded as the final gene target of XLD. Among them, XLD includes 12 kinds of traditional Chinese medicine ingredients, and a total of 200 active ingredients can pass through the blood-brain barrier, of which gancao, Yanhusuo and danshen account for 64% of the total drugs.

#### Screening of GBM-related genes

TCGA (https://www.cancer.gov/about-nci/organization/ccg/research/structural-genomics/tcga) database was launched by the National Cancer Institute (NCI) and the National HumanGenome Research Institute (NHGRI) of the United States in 2005. Through the application of genome analysis technology, especially large-scale genome sequencing, this paper attempts to draw and systematically analyze the genome variation map of all human cancers (the short-term target is 50 kinds of tumors including subtypes). We downloaded 169GBM tumor samples, Genotype-Tissue Expression (https://www.gtexportal.org/home/index.html). This database is different from TCGA and ICGC. On the other hand, GTEx collected tissues from normal people for sequencing, and 1,152 normal brain samples were obtained (Vivian et al., 2017). Using Limma version 3.11 software package (https://bioconductor.org/pack ages/Limma/) to screen the differentially expressed genes between high risk group and low risk group (corrected *p* value and lt; 0.05, fold change ≥ x2), 894 differentially expressed genes were obtained.

### Network analysis

#### Drug-gene targeting network

By using Cytoscape (v.3.8.0) to construct the target network of 166target genes of active components in traditional Chinese medicine that can pass through the blood-brain barrier and 19 genes of drug-disease, and the (CTD) network of compound-target-disease, we can see the core relationship between XLD active component network and molecular targeting prediction target and compound-target-disease (CTD) network. The network analyzer plug-in is used to identify key active components and key candidate targets according to the following criteria: nodes whose values exceed the average of all nodes in the network. The degree value is the number of edges a node has in the network, indicating how many herbs/ingredients/targets a node is associated with. If the degree value of the node is large, it is considered that the node plays a more important role in the network.

#### (PPI) analysis of protein-protein interaction

The 166 target genes of the above drugs and 19 genes of the same drug-disease were introduced into the STRING database to construct the PPI network. STRING database integrates many protein-protein association networks for biological quality control. We chose the core PPI target based on the above-average degree score and the confidence score above 0.9. Used to build PPI networks. Cytoscape is then applied to examine the potential correlation between these genes. The target-target (TT) network between XLD and GBM is constructed using the plug-in CytoNCA of Cytoscape, and the interaction network of gene protein intersection between XLD and GBM is constructed by the plug-in BisoGenet of Cytoscape, and visualized with Cytoscape (Barabási et al., 2011; Al-Harazi et al., 2016; Muthiah et al., 2017).

#### Analysis of GO and KEGG pathway

Then R software was used to deal with the above 169genes and 19 intersecting genes, and clusterProfiler, org. Hs.eg.db, enrichplot and ggplot2, pathview plug-in packages (vision 3.6.2) were used to visualize GO and KEGG, respectively. The *p*-value filtering condition was pvalueFilter <0.05. The corrected *p*-value filtering condition was qvalueFilter <0.05i. In order to further clarify the biological effects of the active components of XLD and the potential mechanism of antagonizing the therapeutic effect of GBM, the selected targets were marked with signal pathways such as cell cycle, p53 and glioblastoma multiforme (KEGG number: map04210, map04115 and map04151) to identify that the interacting genes and networks were mapped using KEGG parsers.

#### Molecular docking

First, the eight key genes were imported into STRING (http://stitch.embl.de/) to accurately obtain the PDB number of these proteins in humans and obtain the two-dimensional structure of the eight components through the PDB number. The 2D structures of XLD active components and their main targetswere downloaded from Pubchem database and PDB database (Rayan, 2009) (http://www.rcsb.org/), respectively, and these molecules were dehydrated and hydrogenated. Then, autoDOCK 1.5.7 software was used to complete the molecular docking analysis. The combination of drug composition and target can be visualized by thermal map, which shows good binding activity, and the lower the binding energy is, the better the docking effect is. MOE software (v.2019.0102) was used to verify the molecular docking of the molecular pairs with the lowest binding energy between drug small molecules and protein macromolecules.

## Results

**FIGURE 1:**
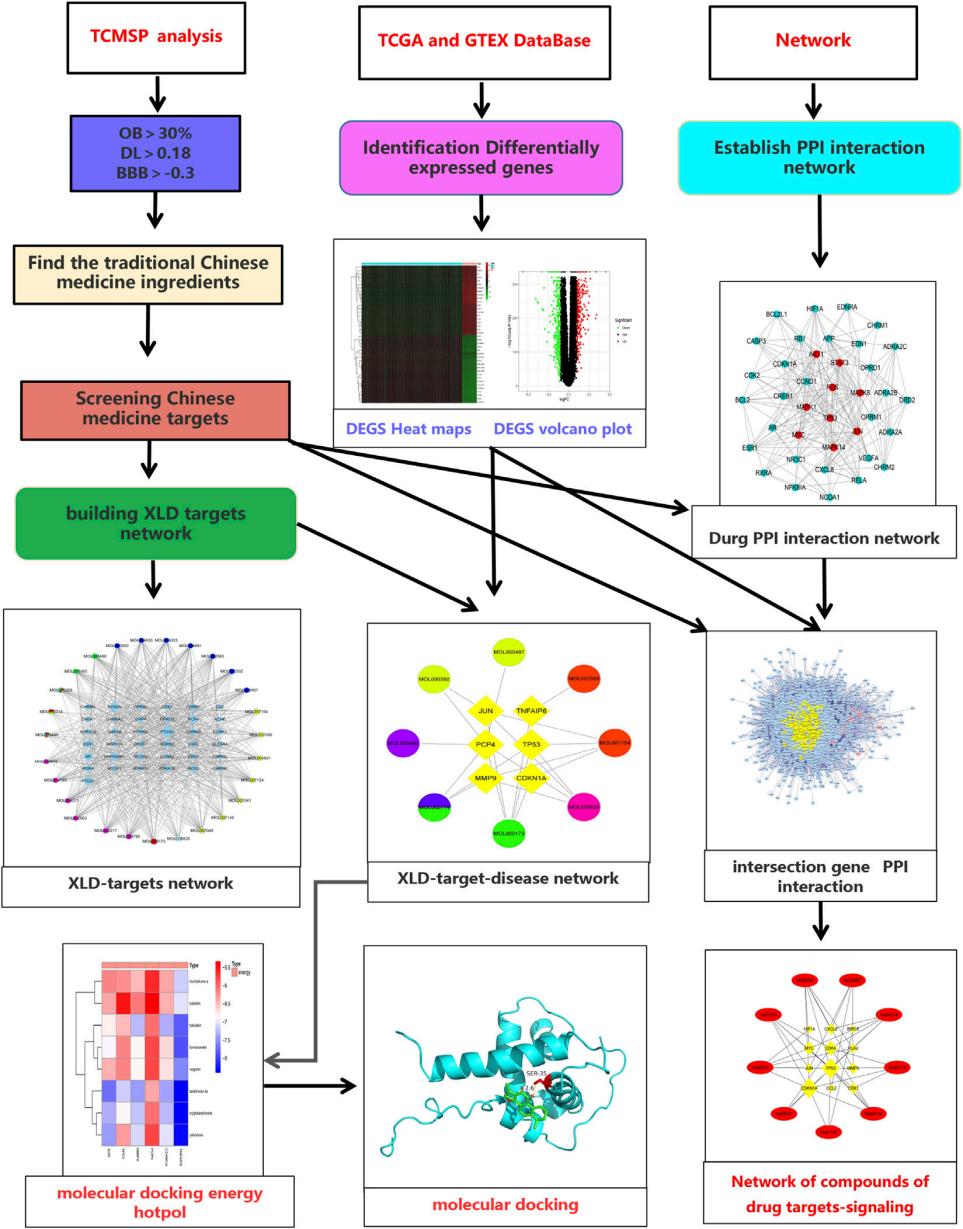
Flow chart of the systematic analysis of XLD for glioblastoma multiforme treatment. TCMSP, Traditional Chinese Medicine Systems Pharmacology Database and Analysis Platform; OB, oral bioavailability; DL, drug-likeness; BBB, blood-brain barrier; TCGA, The cancer genome atlas; GTEX, The Genotype-Tissue Expression; PPI, protein-protein interaction; XLD, Xiaoliu Decoction.

The research process is divided into three stages in order. Figure 1 shows all the processes of system analysis. Firstly, search the active ingredients and targets of XLD formula, and screen differentially GBM expressed genes to build a network. Next, the traditional Chinese medicine related network and glioblastoma multiforme gene network are combined, and the topology analysis is carried out to filter out the required core network. Finally, these targets are analyzed by GO and KEGG to determine the mechanism of HLD against GBM.

### The formula of XLD and the screening of its active ingredients and targets

There are 12 components of XLD, including Baihuasheshecao, baizhi, Baizhu, banxia, Banzhilian, chenpi, Chuanxiong, Chuanxiong, Danshen, Fuling, Gancao, Gouteng and Yanhusuo. To reveal the mechanism of action of XLD in glioblastoma multiforme (the selected drugs can pass through the blood–brain barrier), we downloaded the drug ingredients of XLD from TCMIP and analyzed the ingredients to build a drug network. The initial network contained a total of 346 nodes and 2,626 edges. Then, the final active ingredients were screened using the PubChem database. Among them, 53 were licorice, 41 were Yanhusuo, and 34 were *Salvia miltiorrhiza* Bunge, among which 53 were licorice, 41 were *Glycyrrhiza* uralensis Fisch, and 34 were Salvia miltiorrhiza Miltiorrhizae (Salvia miltiorrhiza Bunge, Radix Salviae Miltiorrhizae). These three types accounted for 71.11% of the total components.

**FIGURE 2:**
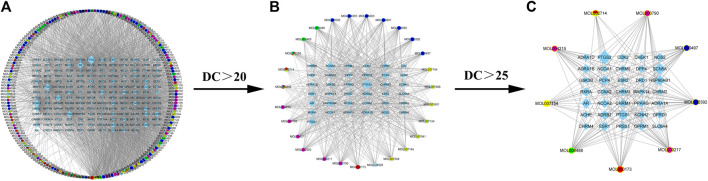
XLD active ingredient network and its molecular targeting relationship. **(A)** XLD formulae network of all active components and molecular targeting network (drug nodes are round and molecular nodes are diamond shaped). **(B)** The 56 drug nodes (round nodes; danshen is yellow, gancao is blue, gouteng is green, yanhusuo is pink, chenpi is red, and a variety of medicines are mixed colors) and 40 molecular nodes (diamond shaped). **(C)**Drug-target network core: The nine drug nodes (round nodes; Salvia miltiorrhiza is yellow, licorice is blue, uncaria is green, Corydalis is pink, tangerine peel is red, and a variety of medicines are mixed colors) and 35 molecular nodes (diamond shaped). XLD, Xiaoliu Decoction; DC, degree centrality.

A compound regulatory network of the relationship between active components and molecules was established with the above-mentioned XLD TCM formula, and an initial network consisting of 346 nodes and 2,626 edges was obtained. Then, topology analysis was applied to filter out the required core network, which contained a total of 44 nodes and 168 edges. This network included nine drug nodes, and 35 molecular nodes (rectangular nodes; the larger the area, the more important the node). The core target network was mapped using Cytoscape (Figure 2).

### Identification of differentially expressed genes in GBM

**FIGURE 3:**
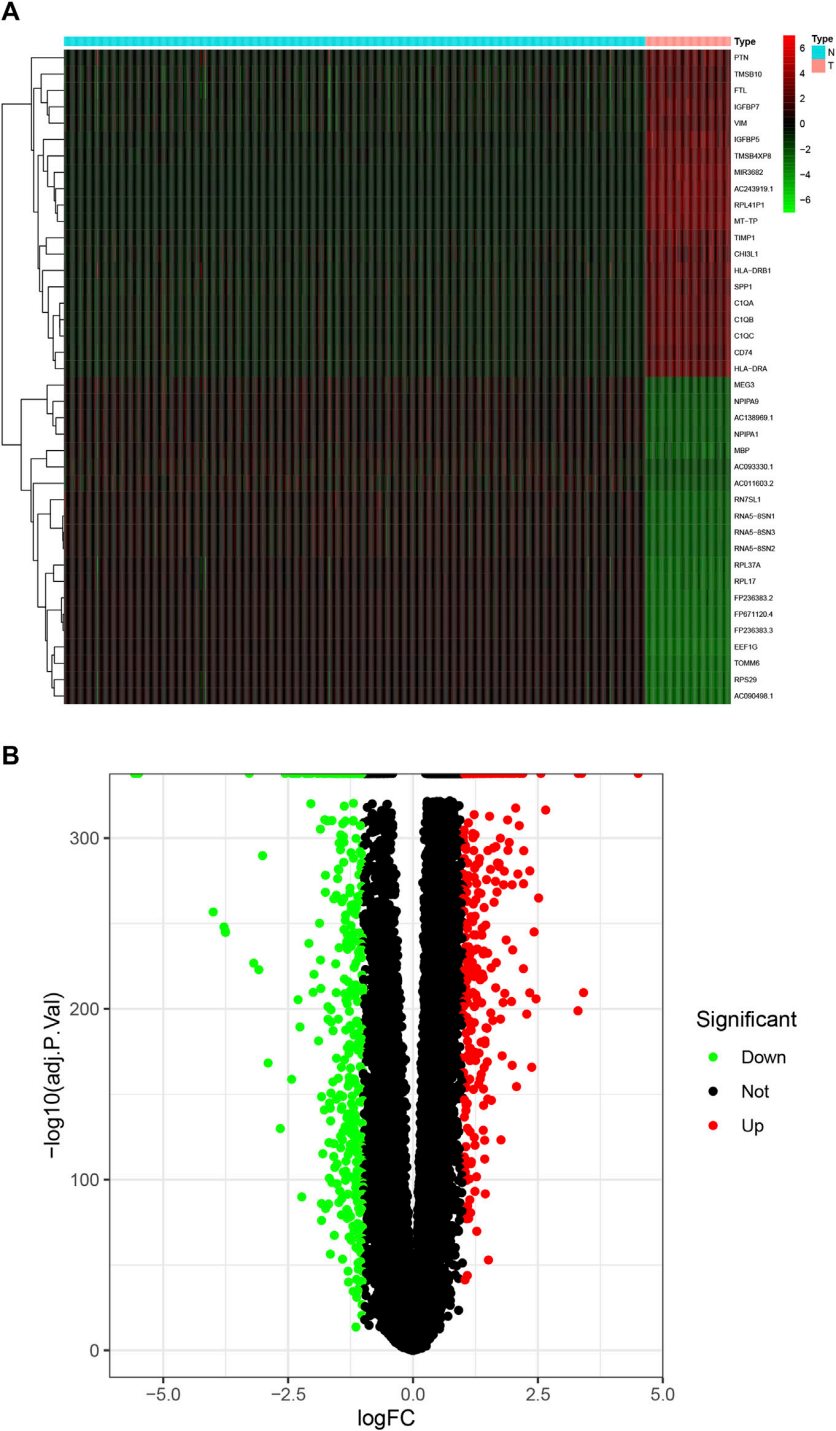
DEGs in GBM. **(A)** Heat maps of the top 50 DEGs in GBM; **(B)** the DEGs volcano plot in GBM. DEGs, differentially expressed genes; GBM, glioblastoma multiforme.

We downloaded the tissue data of GBM patients (169 samples) from the TCGA database and normal brain tissue data (1,152 samples) from the GTEX database. The downloaded data were combined using the limma software package of R software, and differentially expressed genes were screened. Compared with normal brain tissue samples, there were 894 significantly differentially expressed genes in GBM, including 418 upregulated genes and 476 downregulated genes. According to the fold change, *SPP1* (osteopontin) and *HLA-DRA* (HLA class II histocompatibility antigen) showed the greatest change, *MBP* (Myelin basic protein) and *EEF1G* (Elongation factor 1-gamma) showed the highest degree of upregulation, and the first two genes were the most downregulated (screening criteria: FDRFilter <0.05). A heat map (Figure 3A) and a volcano map (Figure 3B) were drawn using the limma software package to visualize the results of differentially expressed genes.

### Construction and analysis of the PPI network

**FIGURE 4:**
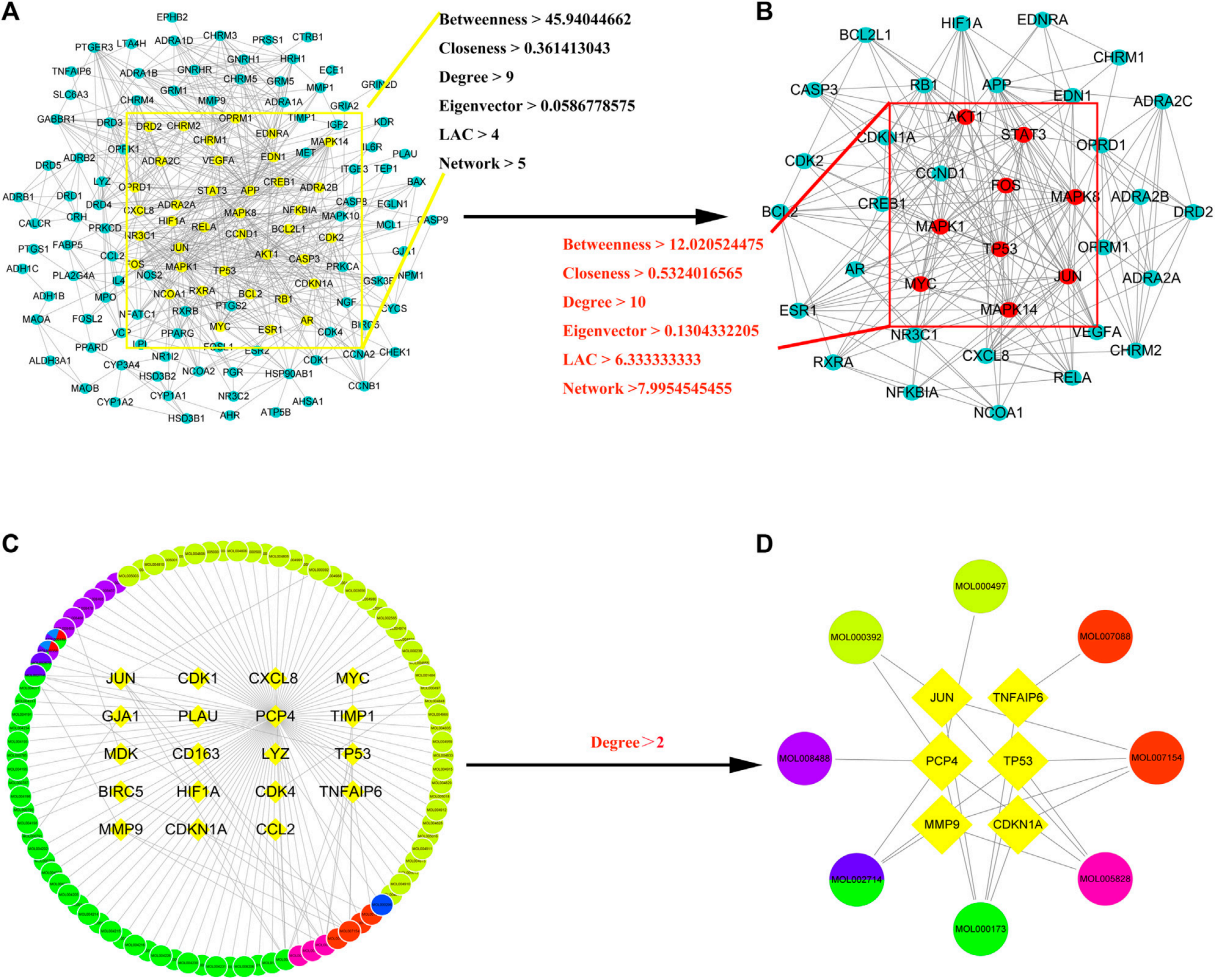
XLD protein interaction core network AND GBM drug-disease-target network for XLD therapy.**(A)** Interaction network of 143 drug target genes, the yellow oval indicates the 38 core genes in **(A)**. **(B)** The 38 core drug target gene interaction network in **(A)**. The red oval indicates the final nine core genes. **(C)** XLD-GBM-target network. **(D)** XLD-GBM-target core network. Common targets for drugs and diseases are indicated by a diamond and drugs are indicated by circles (yellow for danshen, blue for gancao, green for gouteng, pink for yanhusuo, red for Chen Pi and mixed colours for multiple drugs). XLD, Xiaoliu Decoction; GBM, glioblastoma multiforme.

First, a PPI network of the obtained XLD targets was constructed. Importantly, these components can pass through the blood–brain barrier and play an important role in the brain. We obtained a total of 169 gene targets of the effective components in XLD, as shown in Figure 4A. The network map included 143 nodes and 681 edges. Through screening, the core network was obtained, which included 38 nodes and 213 edges. The selection criteria were as follows: Betweenness >45.94044662, centreCloseness >0.361413043, score degree >9 min, Eigenvector >0.0586778575, Lac > 4Command and Network >5 (Figure 4B). *TP53, JUN, MYC, MAPK1, STAT3, FOS* and other genes formed the core of the network.

**FIGURE 5:**
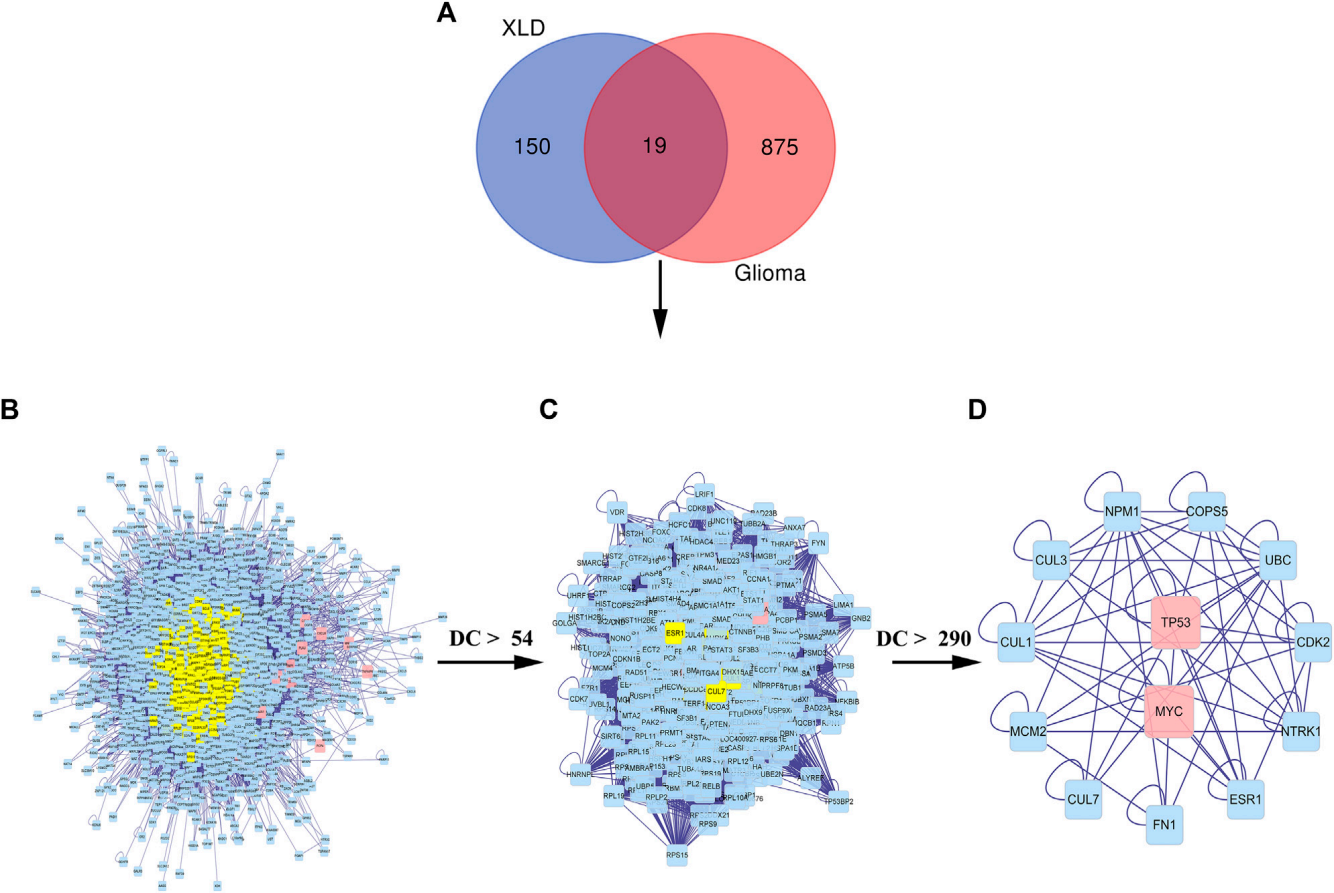
Common PPI network of XLD and GBM targets.**(A)** Wayne diagram of the intersection of the XLD and GBM genes, with a total of 19 shared genes. **(B)** The PPI network of XLD and GBM targets consists of 2,157 nodes and 48,677 edges. **(C)** PPI network of significant proteins extracted from **(B)**. The network consists of 555 nodes and 20,614 edges. **(D)** PPI network of significant proteins in **(C)**. The network consists of 13 nodes and 57 edges. XLD, Xiaoliu Decoction; GBM, glioblastoma multiforme; DC, degree centrality; PPI, protein-protein interaction.

Then, we used the above-mentioned XLD gene target network and the differentially expressed genes in glioblastoma multiforme to construct the drug-disease-target network. As shown in the network diagram in Figures 4C,D there was a one-to-one correspondence between the 95 targets of XLD and 19 drug-disease common genes. The required core network was filtered out by topology analysis. This core network contained eight targets and six gene targets of XLD. The network graph included 14 nodes and 21 edges. Among them, MMP9 was connected to six core targets, and Jun and TP53 were connected to four core targets. The active components and targets of XLD and disease targets were connected and formed a complex network, indicating that XLD can directly or indirectly act on multiple targets in GBM and play an effective therapeutic role. Furthermore, we also predicted the PPI network using 19 target networks common between GBM and XLD components and used the CytoNCA plug-in in Cytoscape to confirm this network. The selection criterion for the complex network (2,157 nodes and 48,677 edges) was as follows: DC > 54 (Figures 5C,D). Finally, the core network with 555 nodes and 20,614 edges was selected.

### GO and KEGG analysis of XLD and glioblastoma multiforme

**FIGURE 6:**
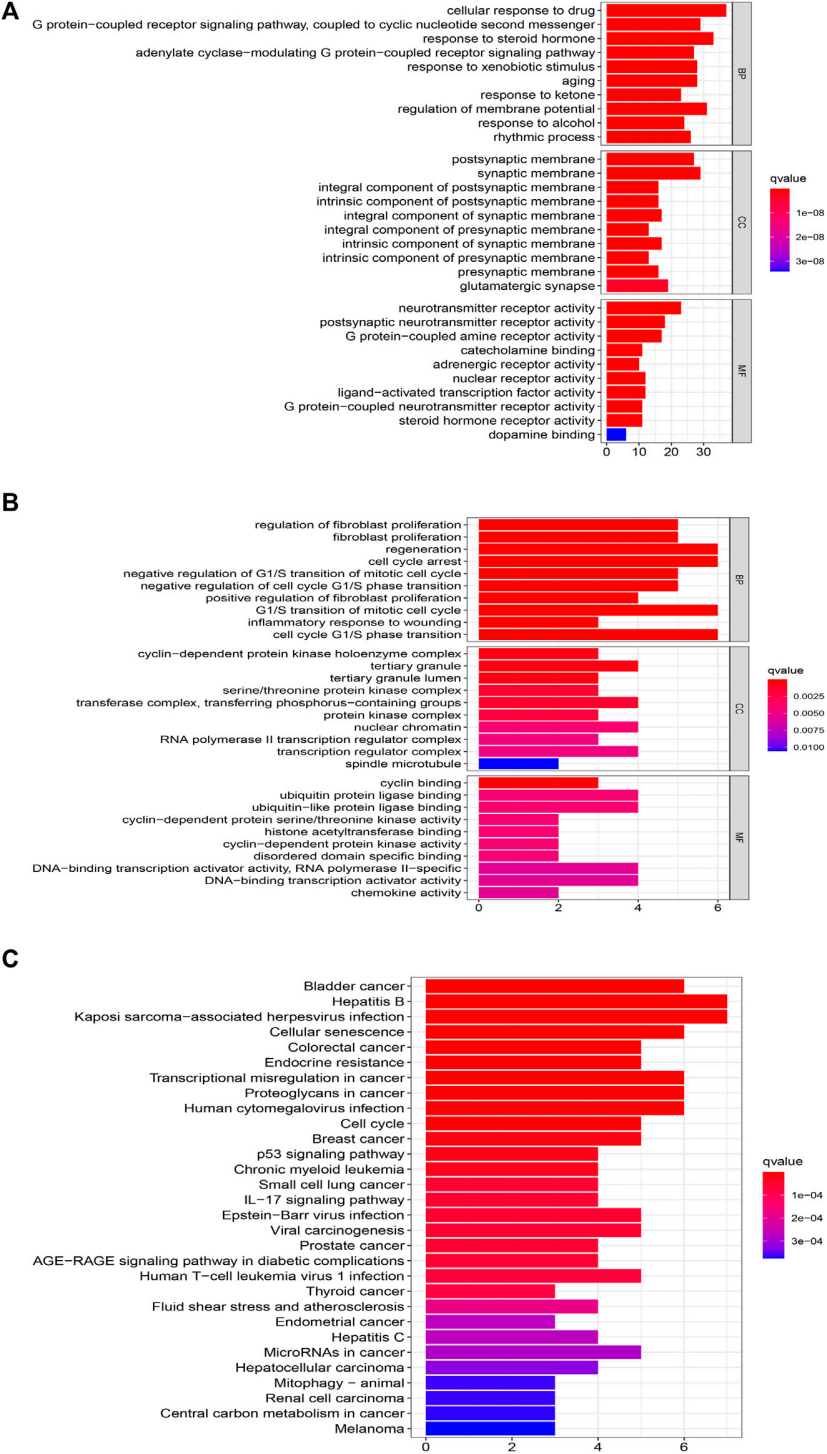
Functional GBM analysis of XLD targets and GBM differential genes.**(A)** GO functional enrichment analysis XLD targets. **(B,C)**GO and KEGG functional enrichment analysis of common targets of XLD and GBM. The size of the bubble indicates the number of enrGBMed genes, and the color indicates qvalue and Pvalue. XLD, Xiaoliu Decoction; GBM, glioblastoma multiforme; GO, Gene Ontology; KEGG, Kyoto Encyclopedia of Genes and Genomes; BP, biological process; CC, cell components; MF, molecular function.

We analyzed the GO and KEGG enrichment of 169 targets of XLD and 19 targets common to glioblastoma multiformes using R software (Figure 6). GO analysis included the cellular component (CC), molecular functional (MF) and biological process (BP). GO analysis showed that the 169 targets were widely distributed but mainly localized to the synaptic membrane, cyclin-dependent protein kinase holoenzyme complex, transcription regulator complex, RNA polymerase II transcription regulator complex, serine/threonine protein kinase complex, dopaminergic synapse, protein kinase complex, outer membrane of organelle, for example. Furthermore, the 169 targets were shown to have a variety of molecular functions, including neurotransmitter receptor activity, G protein-coupled amine receptor activity, nuclear receptor activity, ligand-activated transcription factor activity, steroid receptor activity, RNA polymerase II-specific DNA binding, transcription factor binding, DNA binding, ubiquitin-like protein ligase binding and cyclin-dependent serine/threonine kinase regulatory activity.

Among the 19 common genes of drug and disease, the biological processes were mainly those such as regeneration, cell cycle arrest, negative regulation of G1/S transition of mitotic cell cycle, negative regulation of G1/S phase cell cycle transition, G1/S transformation of mitotic cell cycle, regulation of neuron apoptosis, regulation of cell cycle block, regulation of DNA biosynthesis, positive and negative regulation of pri-miRNA transcription by RNA polymerase II. These mainly involve, for example, the cyclin-dependent protein kinase holoenzyme complex, serine/threonine protein kinase complex, transferase complex, transfer phosphorus-containing groups, complex protein kinases, RNA polymerase II transcriptional regulatory complex, complex transcriptional regulatory agencies.

**FIGURE 7:**
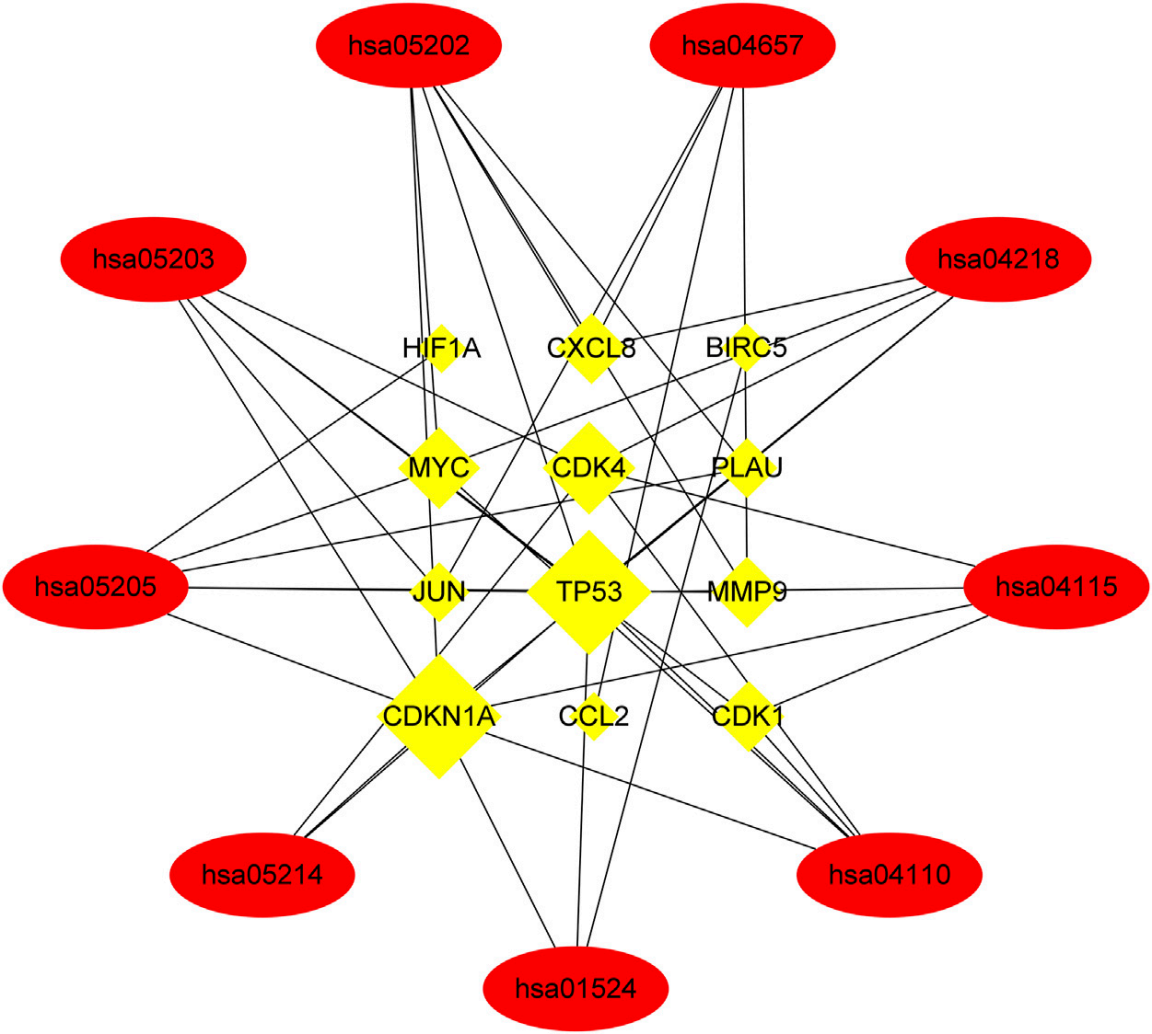
Networks of XLD drug target signalling pathways. A total of nine Kyoto Encyclopedia of Genes and Genomes signaling pathways (elliptical nodes) and 12 genes (rectangular nodes) associated with them were obtained. The size of nodes was proportional to the number of adjacent nodes. hsa, Homo sapiens. Cyclin-dependent kinase 1(CDK1),Interleukin-8(CXCL8),Cyclin-dependent kinase 4(CDK4), Myc proto-oncogene protein (MYC), Cellular tumor antigen p53 (TP53),Cyclin-dependent kinase inhibitor 1(CDKN1A),Urokinase-type plasminogen activator (PLAU), Matrix metalloproteinase-9 (MMP9),Hypoxia-inducible factor 1-alpha (HIF1A),C-C motif chemokine 2(CCL2),Transcription factor AP-1 (JUN), Baculoviral iap repeat-containing protein 5(BIRC5).

Finally, KEGG enrichment analysis was performed on these genes to determine the mechanism of HLT against GBM, and it was found that they are mainly involved in glioblastoma multiforme, cellular senescence, cell cycle and transcriptional misregulation in cancer pathways. To elucidate the overall mechanism of HLT in glioblastoma multiforme, the above steps were repeated during KEGG network construction, which provided a complete mechanism of XLD to understand the pathogenesis of glioblastoma multiforme (Figure 7). Twelve genes (yellow rectangles) and nine KEGG (red rectangles) were obtained by layer-by-layer screening. The larger the area, the more important the genes were in the network. The 12 genes are: *CDK1, CXCL8, CDK4, MYC, TP53, DKN1A, PLAU, MMP9, HIF1A, CCL2, JUN, BIRC5*, seven KEGG passages are: hsa05202, hsa04657, hsa05163, hsa04218, hsa05205 hsa01524, hsa04012, hsa04668, hsa04110, hsa04210.

### Molecular docking analysis

**FIGURE 8:**
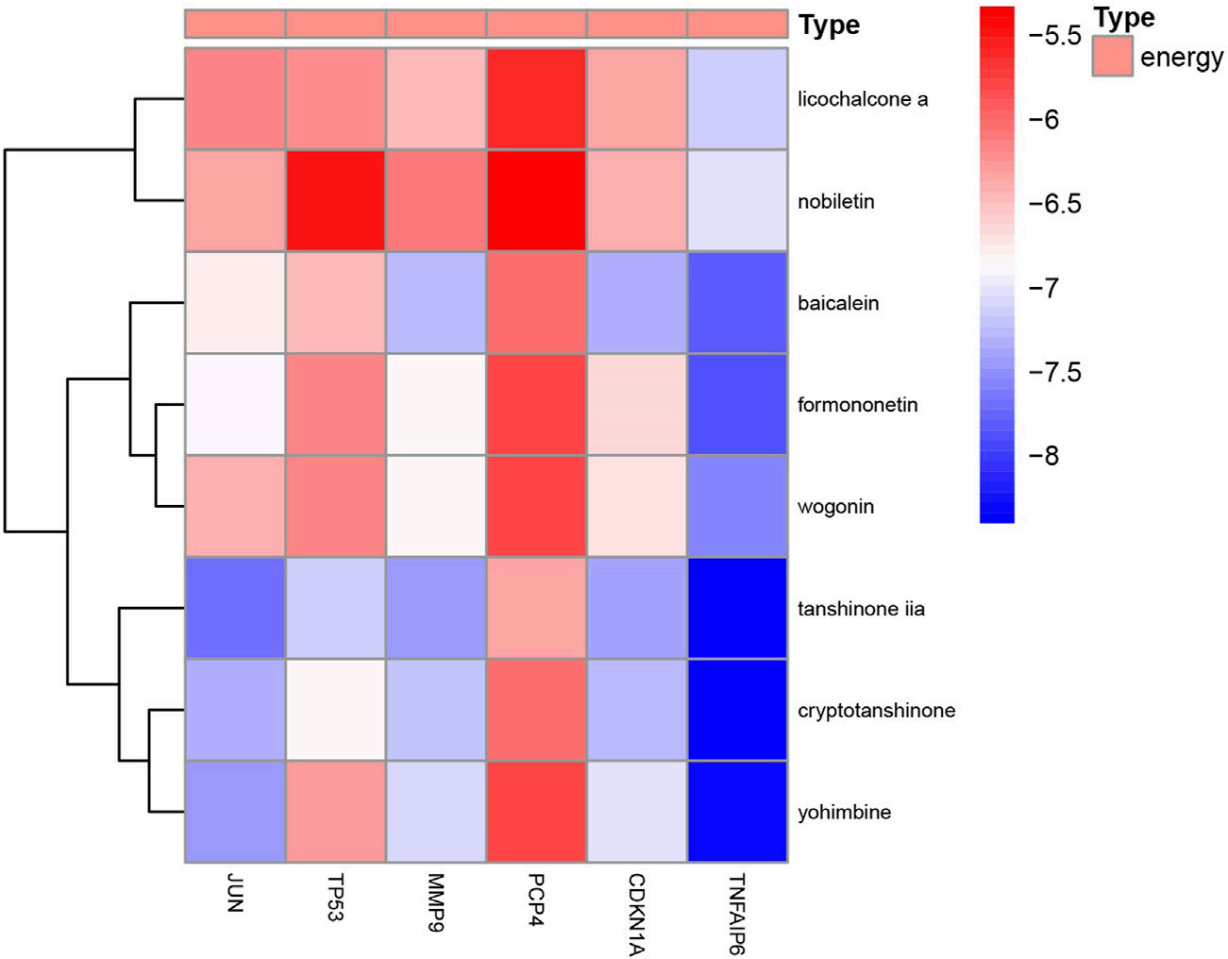
Molecular docking energy heat map. Molecular docking of JUN,TP53,MMP9,PCP4, CDKN1A AND TNFAIP6 proteins in Figure 4 was performed with the corresponding small molecule drugs in the network, and the docking energy was drawn into a heat map. Red represents high docking energy required, and blue represents low docking energy required.

**FIGURE 9:**
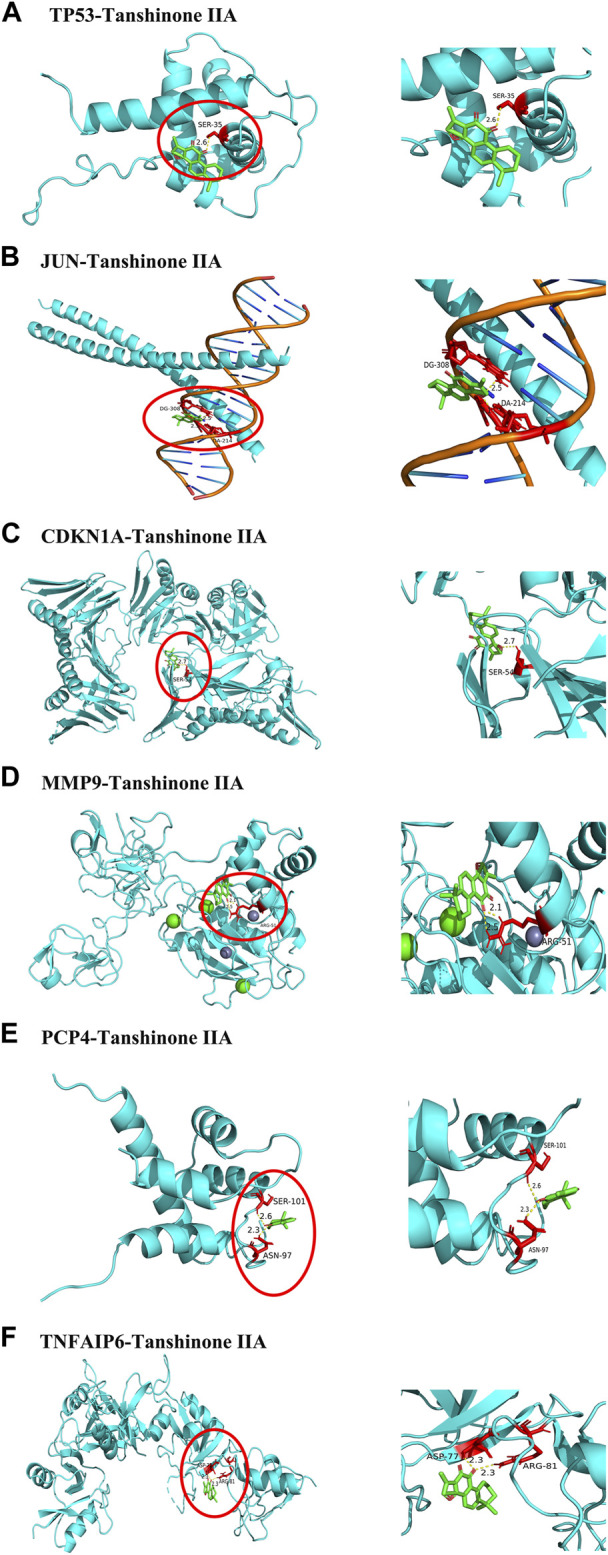
Core protein docking with small molecules. The molecules and drugs with the lowest docking energies are visualized in Figure 8, JUN,TP53,MMP9,PCP4, CDKN1A AND TNFAIP6 proteins and Tanshinone 2A. Small molecule drugs and interacting amino acids are red, proteins are blue, their interactions are yellow dotted lines, and numbers are bond lengths.

To analyze the feasibility of XLD in the treatment of glioblastoma multiforme, we also performed molecular docking analysis on the active components of XLD and the key targets in glioblastoma multiforme. The PDB with the crystal structure of *Jun* was 1JNM, the PDB with the crystal structure of PCP4 was 2N77, the PDB with the crystal structure of MMP9 was 1L6J, the PDB with the crystal structure of TP53 was 2K8F, the PDB with the crystal structure of CDKN1A was 4RJF, and the PDB with the crystal structure of TnFaiP6 was 2PF5. Molecular docking was conducted for all pharmacodynamic components and targets in sequence, and the results were displayed in a heat map (Figure 8). Interestingly, we found that tanshinone 2A had the lowest binding energy among all core drugs. Therefore, we showed the docking diagram of tanshinone 2A with six proteins in Figure 9. These findings provide valuable information for the development of drugs to treat glioblastoma multiforme.

## Discussion

In this study, we systematically explored the potential molecular mechanism of XLD in the brain by performing GO and KEGG pathway enrichment analyses and analyzing the active components and targets of XLD in the PPI network. Furthermore, by comparing the targets of XLD and differentially expressed genes in GBM, we obtained the possible gene targets of XLD in the treatment of GBM. To reveal the potential therapeutic mechanism of the decoction, we collected the components of XLD using TCMSP and TCMID databases and obtained a total of 1,060 active components. Among them, 53 components were found in more than one herbal medicine, and these components shared by different herbs may be the key components contributing to the biological function of XLD. Among the 12 traditional Chinese medicines of anti-tumor decoction, the active ingredients that can pass the blood-brain barrier mainly exist in licorice, Yanhusuo and Salvia miltiorrhiza. Through a literature search, we found that these drugs have a variety of pharmacological properties, such as antioxidant and anti-fibrosis activities (Shin et al., 2008; Li et al., 2010; Fu et al., 2014). Interestingly, our results show that two drugs, licorice and salvia, are the most critical components of the XLD drug network.

Many active ingredients in licorice are related to GBM. One example is licochalcone A, a natural rutin extracted from licorice root with a variety of biological effects, including antioxidant, anti-inflammatory and anticancer activities. Licochalcone A induces caspase-dependent death in GSCs but not differentiated GSCs, normal somatic cells or neural stem cells, and Kuramoto et al. found that licochalcone A caused mitochondrial breakage and decreased membrane potential and ATP production in GSCs, leading to cell death. Moreover, Huang et al. demonstrated that LicA significantly inhibited ADAM9 expression and impaired the migration and invasion activity of human GBM cells (M059K, Umur251 MG, GBM8901) through the MEK/ERK signaling pathway. Lu et al. found that LA effectively inhibited the growth of U87 GBM cells by inducing G0/G1 and G2/M cell cycle arrest, an effect that was attributed to reduced cyclin and cell cycle-dependent kinase mRNA and protein levels (Kuramoto et al., 2017; Huang et al., 2018; Lu et al., 2018). Lupiwighteone (Lup) is a natural isoflavone extracted from licorice. Ren et al. found that Lup has a concentration-dependent and time-dependent effect on the growth of SH-SY5Y cells. Lup induced G2/M phase arrest and significantly reduced the protein expression of cyclin B1/D1 and cyclin-dependent kinase (CDK)-1 and 4–6. Furthermore, Lup altered mitochondrial membrane potential and increased the production of intracellular reactive oxygen species (ROS) (Ren et al., 2015). The anticancer effects on SH-SY5Y cells provide a scientific basis. Formononetin is a recently identified type of TCM isolated from licorice that has anti-tumor activity. Studies have shown that formononetin combination therapy reverses adriamycin-induced epithelial-mesenchymal transformation (EMT) of tumor cells and prevents EMT by inhibiting HDAC5, thereby enhancing the therapeutic effect of adriamycin on GBM cells. Zhang et al. also found that formononetin combined with temozolomide (TMZ) enhanced the expression of Bax, cleaved caspase-3 and cleaved caspase-9, decreased the expression of Bcl-2 and promoted the apoptosis of GBM cells. Moreover, the combination therapy downregulated the expression of matrix metalloproteinase-2 (MMP-2) and MMP-9 and inhibited the migration of GBM cells (Liu et al., 2015; Zhang et al., 2018; Ni et al., 2019). The anticancer drug coumarin has attracted increasing attention in recent years, and glycyrol is the most important representative component in coumarin, which is an active ingredient in licorice. Recently, Lu et al. showed that a glycyrol/butyric acid mixture had the strongest inhibitory effect on colorectal cancer cells by enhancing the activation of caspase-3. Benzofuran, isopentene and methoxy groups in glycyrol play a key role in its anticancer activity. Additionally, furan groups further enhance its anticancer activity. Molecular targeted therapy for non-small cell lung cancer (NSCLC) has shown good efficacy. T-Lymphokine-activated killer cell-derived protein kinase (TOPK) is overexpressed in many cancer types, including NSCLC, and is considered to be an effective target for the treatment of lung cancer. Lu et al. demonstrated that glycyrol binds to TOPK and inhibits its kinase activity, resulting in activation of the apoptosis signaling pathway and inhibition of lung cancer cell growth. Moreover, Xu et al. also found that glycyrol induces G0/G1 phase cell cycle arrest, promotes the activation of c-Jun n-terminal kinase (JNK)/p38 mitogen-activated protein kinase (MAPK) and induces caspase-dependent apoptosis, accompanied by the activation of adenosine monophosphate-activated protein kinase (AMPK) (Xu and Kim, 2014; Lu et al., 2019; Lu et al., 2020). Together, these results demonstrate the anti-tumor activity of glycyrol, highlighting its potential for the treatment of cancer.

There are 34 active ingredients in *Salvia miltiorrhiza*. *Salvia miltiorrhiza* dispels blood stasis and relieves pain, promotes blood circulation and relieves menstrual pain. *Salvia miltiorrhiza* contains fat-soluble and water-soluble components. Most of the fat-soluble components are conjugated quinones and ketones, such as tanshinone I, tanshinone IIA, tanshinone IIB and cryptotanshinone. The water-soluble components include Danshensu, Danshen acid A, protocatechuic acid and protocatechuic aldehyde. Among them, tanshinone, cryptotanshinone and tanshinone have all been shown to have effects on GBM cells (Wang et al., 2007; Tang et al., 2010; Yang et al., 2014; Ren et al., 2015; Ding et al., 2017; You et al., 2020; Liu et al., 2021). Furthermore, they also have anti-diabetes, anti-inflammation, antioxidant and anti-cancer therapeutic effects (Wang L. et al., 2017; Maione et al., 2018; Jia et al., 2019; Li et al., 2021; Liu et al., 2021).

In the analysis of XLD targets using the PharmMapper database, 169 targets were obtained, which were found to be closely related to 200 active compounds in XLD. Among them, JUN, TP53, MYC, FOS and STAT3 are closely related to the active components of XLD and associated with the pathophysiology of GBM and the mechanism of tumorigenesis and development.

To understand the specific regulatory mechanism of XLD, 169 targets of XLD were systematically screened by GO and KEGG analyses, which revealed the multi-pathway targets of XLD. GO analysis shows that the 169 targets are widely distributed throughout the nervous system and may be involved in a variety of biological processes, such as cell cycle regulation, transcriptional regulation and negative regulation of apoptosis. Furthermore, KEGG analysis of XLD and GBM intersection genes showed that they were mainly involved in the signaling pathways of GBM with a wide range of anticancer effects, including participation in central carbon metabolism in cancer.

Transcriptional misregulation in cancer, cell cycle, p53 signaling pathway and platinum drug resistance, for example. By comparing the results of GO and KEGG analyses, we found that their results were complementary and interrelated, which facilitates a more comprehensive understanding of XLD. In summary, components in XLD that can pass through the blood–brain barrier may have a therapeutic effect on tumors and GBM through a variety of different pathological mechanisms.

GBM is one of the most aggressive and incurable diseases characterized by high tissue heterogeneity and rapid transformation from low-grade (I–II) to high-grade (III–IV). Approximately 95% of low-grade GBM progress into high-grade tumors; less than 3% of these patients are still alive 5 years after diagnosis (Bastien et al., 2015; Tamimi and Juweid, 2017; Ostrom et al., 2020). At present, surgery, radiotherapy and TMZ chemotherapy are first-line treatments for GBM, and the degree of tumor resection affects the prognosis of GBM patients (Bastien et al., 2015; Tamimi and Juweid, 2017; Ostrom et al., 2020). Unfortunately, complete removal of the tumor cannot be achieved because of the infiltrating nature of GBM cells. Therefore, the realistic goal of neurosurgeons is to remove 90% of the tumor without causing neurological defects associated with surgery. Regarding chemotherapy, it is non-specific and has harmful effects on cells and tissues, including nausea, fatigue, significant myelosuppression, thrombocytopenia, severe infection and myelodysplastic syndrome (Malmström et al., 2017; Schreck and Grossman, 2018).

With a better understanding of genes, new treatments have been developed. For example, gene therapy can be used to inhibit the carcinogenic properties of tumor cells (Caffery et al., 2019; Hossain et al., 2020). Gene therapy in cancer involves introducing tumor suppressor or growth regulatory genes into the tumor [56]. Because conventional therapy cannot overcome treatment resistance, gene therapy can be used to manipulate the genetic composition of tumor cells to provide therapeutic benefits. However, these gene therapies must pass through the blood–brain barrier to achieve the therapeutic effect. To improve the transmission of these therapies, delivery vectors such as viral vectors, polymeric nanoparticles and non-polymeric nanoparticles have been studied. However, a meta-analysis found that these studies were not statistically significant (*p* = 0.13) (Artene et al., 2018). Furthermore, viral therapy did not statistically improve progression-free survival. Therefore, gene therapy with viral agents alone may not be a feasible treatment in HGG. In immunotherapy, monoclonal antibodies have high affinity and specificity in targeting growth factor receptors, such as PDGFR, VEGFR and EGFR. One challenge with monoclonal antibodies is that because of their large size, they may not easily pass through the blood–brain barrier. Therefore, to overcome this limitation, monoclonal antibodies can be connected to the surface of nano-carriers through the pre-adsorption process to prevent the formation of biomolecule crowns (Tonigold et al., 2018; Loureiro et al., 2020). These treatments are not only expensive, but they also need to pass through the blood–brain barrier, and the efficacy should be considered.

The discovery of plant-derived bioactive compounds as new therapies may provide a therapeutic advantage in GBM research. Approximately 60% of clinically approved anticancer drugs on the market come from medicinal plants (Yool and Ramesh, 2020). Their multi-target, high selectivity, reduced multidrug chemical resistance, cost-effectiveness and minimal side effects make them valuable potential therapies, especially when used in combination with current treatment strategies (Rayan et al., 2017). TMZ used in combination with phytochemicals, such as thymoquinone and cannabinoids, has been shown to enhance the anticancer effect in preclinical models (Dumitru et al., 2018; Pazhouhi et al., 2018). The introduction of the big data concept and the continuous development of pharmacology provide an opportunity to analyze the relationship between drugs and molecular targets (Zhang et al., 2013). Network pharmacology provides a useful approach to explore the regulation of multi-channel signaling pathways, improve the efficacy of drugs and the success rate of clinical trials, and reduce the cost of drug development. Chinese herbal medicines and plant ingredients are positive prospects in the treatment of a variety of complex diseases (Sang et al., 2020). Network pharmacology has been widely used to study the biological mechanism of some prescriptions and components of TCM (Zhu and Hou, 2020). Therefore, we used network pharmacology to understand the biological mechanism by which XLD targets GBM at the molecular level.

In China, XLD is widely used in the treatment of tumors, including GBM. Therefore, comprehensively studying the specific mechanism of XLD in the treatment of tumors, especially GBM, is necessary. This information can help us better understand the regulatory mechanism of drugs in tumors, elucidate the mysteries of TCM and identify new research directions. To address the shortcomings of current gene therapy and immunotherapy regimens and provide novel and effective methods. Therefore, we used R software to study the biological process of related genes. The results showed that XLD regulates the proliferation of tumor cells by affecting the following biological processes: lipopolysaccharide response, phospholipase C-activated G protein-coupled receptor signaling pathway, radiation response, oxidative stress response, transcription initiation of RNA polymerase II promoter, DNA template transcription, hypoxia response and others. Because these regulatory mechanisms are closely related to the occurrence and development of GBM, we hypothesize that XLD may affect the above pathways to regulate GBM cell proliferation, metastasis and other tumor biological processes. To understand the mechanism by which XLD regulates genes in GBM, the expression data of GBM tumor tissues and normal brain tissues were downloaded from TCGA and GTEX databases, and the differentially expressed genes were obtained by R software. The drug-disease-target PPI network was constructed between the target genes of XLD and the differentially expressed genes in GBM. We found that *TP53* and *Jun* were located in the core, indicating that they may be a key hub mediating the effects of XLD on a variety of pathological mechanisms in GBM. Mutations in the tumor suppressor gene *TP53* occur in a variety of cancers, including GBM, which usually lead to a loss of TP53 function and several transcriptional changes, promoting the development of tumors. Approximately 30% of glioblastomas carry *TP53* mutations. TP53-dependent cell cycle arrest has been shown to be involved in mediating the sensitivity of chemotherapy. Additionally, TP53 has been identified as the main regulator of CSC self-renewal, differentiation and tumorigenic potential in glioblastoma (Moreno et al., 2007; Zheng et al., 2008; Varna et al., 2009; Rivlin et al., 2011). However, no drug targeting TP53 has been developed, and only two compounds targeting mutant p53 are currently being investigated in clinical trials, including APR-246 in phase II trials and COTI-2 in phase I trials (Maslah et al., 2020; Synnott et al., 2020). To further analyze the feasibility of XLD in the treatment of GBM, we performed molecular docking analysis and found that many active components have a high affinity for the core targets in GBM. The components of XLD can each bind to the target molecules. Our analysis of XLD and glioblastoma multiforme system revealed that JUN, TP53, MMP9, PCP4, CDKN1A and TNFAIP6 proteins are the core proteins of XLD in the treatment of glioblastoma multiforme, and KEGG enrichment analysis revealed that these proteins regulate the development of glioblastoma multiforme mainly by regulating cellular senescence, transcriptional misregulation in cancer and affecting the cell cycle. The KEGG enrichment analysis revealed that these proteins regulate the development of glioblastoma multiforme by regulating cellular senescence, transcriptional misregulation in cancer and affecting the cell cycle. Interestingly, we docked the core proteins to the core small molecule drugs and found that tanshinone iia has the lowest docking energy to the core proteins, thus, tanshinone iia may become a small molecule target drug for the treatment of glioblastoma multiforme.

There are still some shortcomings in this study that need to be investigated in depth in the next step. Firstly, only a computer-based exploration of the mechanism of XLD for the treatment of glioblastoma multiforme was used. Next, we need to further confirm the therapeutic effect of XLD on GBM through basic experiments using the core small molecule drugs in our conclusions. In addition, the optimal dose of XLD for treating patients with GBM needs to be determined. In conclusion, we have now obtained a possible mechanism for the treatment of GBM with XLD through a network pharmacological analysis and hope that drugs including tanshinone iia will become a new and promising targeted chemotherapeutic agent. It remains to be further elucidated whether it can be a new adjuvant therapy. In China, XLD has been used clinically with relatively few adverse effects, and therefore it is promising to be used in GBM-related clinical trials.

## Data Availability

The original contributions presented in the study are included in the article/supplementary material, further inquiries can be directed to the corresponding authors.

## Abbreviations

CTD, network of compound-target-disease; GBM, glioblastoma multiforme; GTEx, Genotype-Tissue Expression; LGG, low grade glioma; TCGA, the Cancer Genome Atlas; TCMSP, traditional Chinese Medicine system Pharmacology Database; XLD, Xiaoliu decoction.
